# A Note on the Fractal Behavior of Hydraulic Conductivity and Effective Porosity for Experimental Values in a Confined Aquifer

**DOI:** 10.1155/2013/356753

**Published:** 2013-12-09

**Authors:** Samuele De Bartolo, Carmine Fallico, Massimo Veltri

**Affiliations:** Department of Civil Engineering, University of Calabria, 87036 Rende, Italy

## Abstract

Hydraulic conductivity and effective porosity values for the confined sandy loam aquifer of the Montalto Uffugo (Italy) test field were obtained by laboratory and field measurements; the first ones were carried out on undisturbed soil samples and the others by slug and aquifer tests. A direct simple-scaling analysis was performed for the whole range of measurement and a comparison among the different types of fractal models describing the scale behavior was made. Some indications about the largest pore size to utilize in the fractal models were given. The results obtained for a sandy loam soil show that it is possible to obtain global indications on the behavior of the hydraulic conductivity versus the porosity utilizing a simple scaling relation and a fractal model in coupled manner.

## 1. Introduction

The scale concept is strongly related to parameters characterizing flow and transport in porous media, mainly to hydraulic conductivity, for which the scale behaviour was widely verified in the literature [1–11], and to the role of this related to the effective porosity, which strongly influences flow in porous media [12–22]. The causes of the scaling behaviour are generally attributed to the medium heterogeneity [7, 23, 24]. Specifically, it was noted that different scales (laboratory scale, field scale, regional scale, and so on) could be considered according to the specific problem investigated and to the type or the particular method of measurement considered. Furthermore, at a different scale, the manner in which the heterogeneity influences the scale behavior is generally different, mainly the shape and size of pores, from small scales and their continuity from larger ones [12, 16, 18, 19, 22, 25–27]. In this framework the effective porosity, with other parameters such as the tortuosity and the pore network connectivity, plays a fundamental role with regard to the water flow in the porous medium. In any case the scale behavior of the effective porosity is a topic that should again be well characterized in the proper measurement scale and in the other contexts of the scales involving the measurement of the same hydraulic conductivity [28–37]. Therefore, below we will refer exclusively to that parameter (*φ*).

To describe the scale behavior the majority of the studies on this topic consider power type laws. However, even this aspect requires further investigation and clarification, especially considering that often the data set of the parameter in question is composed of subsets achieved by different measuring methods, which generally involve aquifer volumes of different sizes and hence different scales. The relative reference scale varies also for increasing aquifer volumes, as a result of the different ways in which the heterogeneity influences the phenomenon. This occurs in all real aquifers, many of which are often considered homogeneous in the absence of stratifications or obvious differences related to geological hazards. The assumption for the porous medium of the scale homogeneity is well suited to the hypothesis of self-similarity, with respect to which the fractal models can be taken into account [22, 36].

With reference mainly to the hydraulic conductivity, many authors [9, 38, 39] took into account the scale-invariant behaviour of a well-defined measurement range and addressed the scaling study and the description of the hydraulic conductivity and the effective porosity. Specifically, the relationship between these two quantities in terms of measurement, in most cases represented by laws of power type and fractal models, has often concerned well-defined ranges of scale in the context of the measures taken. However, some problems still remain open regarding the definition of scaling cut-off limits, if these exist, with respect to which the measurement scale adopted may well be considered representative. This aspect can be examined in terms of simple-scaling and multiscaling behavior but does not exclude the possibility of interpretation of these scaling behaviors by other laws, also different from those of the power type [40].

The simple and multiscaling behavior of hydraulic conductivity in the last two decades was put in relation both in terms of porosity and in terms of measurement scale. The description of this behavior in terms of power law, among many works in the literature, can be traced to the early experiences of Jacquin and Adler [12] and Muller and McCauley [41] on the generalization of the Kozeny-Carman equation in terms of fractal geometry (geometry of porous geological structures) if the process is considered in the pore scale, until recent studies provided by Xu and Yu [36]. While for the scale of measurements (including aquifer tests) reference works can be traced to those of Neuman [4], Rovey II and Cherkauer [5], Guimerà et al. [6], Schulze-Makuch, and Cherkauer [8]. As part of the relationship between hydraulic conductivity (*k*) and porosity (*φ*) the basic equations had also a review in terms of pore-space fractal geometry assumption [22], achieving good results for nongranular systems (fiber mats and vesicular rocks). Developments related to further scales (laboratory and field) can be considered, such as those of Giménez et al. [16], Pape et al. [18], Giménez et al. [19], and Regalado and Muñoz-Carpena [21]. Some considerations can be made for the effective porosity, which is a structural parameter of fundamental importance for the description of the water flow in porous media. However, the scaling behaviour of the effective porosity still requires further investigation and details in order to consider the topic sufficiently characterized, as pointed out by numerous studies examining also the relationship between this parameter and hydraulic conductivity and the context of the scales involving the measurement of these two parameters [28–37].

The use of fractal models can provide important guidelines on the determination of the physical scaling relative to the measurement scale. This specifically concerns the functional link between porosity and scale and between hydraulic conductivity versus porosity, which is equivalent to the functional link between hydraulic conductivity and scale. In any case one should note that the parameter estimations concerning the fractal models are not immediate, both in terms of knowledge of the fractal dimension itself and of other geometrical and/or physical parameters linked to it. This aspect is also related to the fact that most of the known fractal models were verified for homogeneous porous media. The use of empirical relationships, as power laws of sample-scaling type, can provide an early indication about the overall trends of the functional link between the hydraulic conductivity and porosity.

In this work, on the basis of data sets obtained by laboratory and field measurement methods, the scaling behaviour analysis of the hydraulic conductivity and effective porosity was performed for the relative characteristic measurement scales relative to a confined aquifer, characterized by a sandy loam porous media and considered homogeneous for the absence of stratification. This analysis, on the basis of a relation of a simple-scaling behavior, coupled with other fractal models such as suggested by Xu and Yu [36], Jacquin and Adler [12], and Muller and McCauley [41], shows that the scale index, for this specific soil type, is connected with the fractal dimension, which is determined directly considering the values obtained by the scaling law utilized. This analysis shows a new interpretation of the maximum pore size of the porous medium, starting from the considerations provided by Xu and Yu [36].

This direct approach, allowing the hydraulic conductivity to be related to the effective porosity, can be utilized to give a comparison parameter with the several fractal models used to describe the scaling behavior of the hydraulic conductivity and the effective porosity.

## 2. The Role of Fractal Geometry in the Framework of Porosity and Hydraulic Conductivity

The scaling behavior between the saturated hydraulic conductivity (*k*) and the effective porosity (*φ*) can be represented by the following power-law relationship [12]:

(1) $$\begin{array}{c} k\approx \varphi^{\mu }, \end{array}$$

where *μ* is the scale crowing index and *φ* depends on the measurement scale (*s*) and the pore size (*λ*). This functional relation (1) is often associated with fractal models and therefore *μ*, a function of the fractal dimension of pores (*D*_*f*_), can be proportional to the rate in which *D*_*f*_ appears. For example, according to Jacquin and Adler [12], this result is proportional to the ratio (4 − *D*_*f*_)/(2 − *D*_*f*_) for a model where the largest pores provide the pore-space connectivity. Other authors gave different function relations of the *μ* scale index, for example, Muller and McCauley [41], Korvin [42], and Giménez et al. [16]. In particular, in Muller and McCauley [41] this exponent is related to (4 − *D*_*f*_)/*D*_*f*_ and is very close to that obtained by Jacquin and Adler [12].

From the theoretical point of view, the fractal dimension appears as one of the parameters that can be used in order to describe the porous medium. As such, it should occur in the expression of hydraulic conductivity, which is the only function of the geometry of the problem. In fact, according to Ahuja et al. [43] and Jacquin and Adler [12], the relation (1) is not retained sufficient in terms of cut-off limits and therefore the same may be generalized in the following functional relation:

(2) $$\begin{array}{c} k=f\left(\varphi ,D_{f},\hat{D},\ldots \right), \end{array}$$

where $\hat{D}$ is a spreading dimension, which involves a more complex scaling range. For these latter reasons the assessment of $\hat{D}$, with each associated scaling behaviour, does not result immediately. In fact, as is well known in the literature [44], these scaling ranges are generally narrow, because these are determined by the type of fractal model used and by the same fractal analysis performed in two- and three-dimensional supports of the measurements (see also [41]). *A fortiori*, without predefining a fractal model, it is possible to perform a direct scaling analysis to obtain the same scale exponent, without explicitly stating the fractal dimension. This last approach, already considered in the literature [10, 11], is well suited to interpreting the experimental results, considering the possible presence of simple and multiscaling behaviours of the investigated parameter. Therefore, relation (1) can be expressed by the following empirical power law:

(3) $$\begin{array}{c} k\approx a\varphi^{m}, \end{array}$$

where *a* is a coefficient depending on the specific porous media and *m* is a general scale crowing index.

In terms of grain size distribution, the relation (3) is also clarified by the classical permeability-porosity relation of Kozeny [45], Carman [46], applied in various fields, such as groundwater flow, water/oil reservoirs, and so on. Recently Xu and Yu [36] developed a new form of the Kozeny-Carman relation for homogeneous porous media by fractal geometry, considering an expression of the porosity, in terms of the fractal dimension (*D*_*f*_) by exactly self-similar fractal geometry supports, namely, the Sierpinski carpet and gaskets, according to what is introduced by Muller and McCauley [41]. In this case the porosity relation, implicitly written in (1) and (3), is clarified by the following relation [47]:

(4) $$\begin{array}{c} \varphi ={\left(\frac{\lambda_{\min}}{\lambda_{\max}}\right)}^{d_{E}-D_{f}}, \end{array}$$

where *λ*_min⁡_ and *λ*_max⁡_, respectively, are equal to the minimum and maximum diameters of the pores characterizing the saturated porous medium, *d*_*E*_ is the Euclidean dimension, which is equal to 2 and 3 in the two- and three-dimensional spaces, respectively. For the theoretical analysis, as suggested by Xu and Yu [36], according to (4), the pore area fractal dimension *D*_*f*_ can be determined by

(5) $$\begin{array}{c} D_{f}=d_{E}-\frac{\ln\varphi }{\ln\left(\lambda_{\min}/\lambda_{\max}\right)} \end{array}$$

while *λ*_min⁡_ ≪ *λ*_max⁡_ must be satisfied for fractal porous media. This latter aspect plays a fundamental role in the determination of the fractal dimension for the specific soil type and for the specific grain size distribution. The model of Xu and Yu [36] is essentially based on the characterization of the fractal dimension, *D*_*f*_, and the tortuosity fractal dimension, *D*_*T*_ [23, 35, 37, 48–50]. In this model the relationship between the hydraulic conductivity and the effective porosity is expressed by the following relation:

(6) $$\begin{array}{c} k=C_{f}{\left(\frac{\varphi }{1-\varphi }\right)}^{(1+D_{T})/2}\lambda_{\max}^{2}, \end{array}$$

where the coefficient *C*_*f*_ is equal to

(7) $$\begin{array}{c} C_{f}=\frac{{\left(\pi D_{f}\right)}^{(1-D_{T})/2}{\left[4\left(2-D_{f}\right)\right]}^{(1+D_{T})/2}}{128\left(3+D_{T}-D_{f}\right)}. \end{array}$$

Xu and Yu [36] in the discussion of their model represented by (6), on the basis of a simple arrangement of solid particles for the maximum pore, give the following relation that allows determination of the maximum pore diameter, namely,

(8) $$\begin{array}{c} \lambda_{\max}=d\sqrt{\frac{\varphi }{1-\varphi }} \end{array}$$

which is expressed as a function of particle diameter *d* and porosity.

Relation (8), which is the basis of the Xu and Yu [36] model, assumes precise values from the experimental point of view, which can be investigated in the grain size distribution context of the porous medium considered. However, as will be shown later in this study, relation (8) is difficult to fit not considering the self-similarity space on the measurement scale of the effective porosity and not taking into account all the set of normalized values represented by the maximum pore distribution expressed in the same equation (8). The experimental evidence, in consideration of the hypothesis of self-similarity in the scale of *φ*(*s*, *λ*), as will be shown for the case considered here, showed that the diameter *d*, taken as representative size of the particle, can be *d*_10_, as well as being considered in many empirical and semiempirical formulas available in the literature [45, 46, 51–55].

Regarding the tortuosity dimension, *D*_*T*_, Xu and Yu [36] give the following relation for tortuous streamtubes in porous media:

(9) $$\begin{array}{c} D_{T}=1+\frac{\ln\bar{\tau }}{\ln\left(L_{0}/\bar{\lambda }\right)}, \end{array}$$

where the average tortuosity $\bar{\tau }$ is given by the results of Yu and Li [56] expressed by

(10) $$\begin{array}{c} \bar{\tau }=\frac{1}{2}\left[1+\frac{1}{2}\sqrt{1-\varphi }+\sqrt{1-\varphi }\frac{\sqrt{{\left(1/\sqrt{1-\varphi }-1\right)}^{2}+1/4}}{1-\sqrt{1-\varphi }}\right] \end{array}$$

and *L*_0_ can be considered the upper cutoff or the upper limit of self-similarity, proportional to *λ*_min⁡_, while $\bar{\lambda }$, namely, the average pore-capillary size, is given by [47]

(11) $$\begin{array}{c} \bar{\lambda }=\frac{D_{f}\lambda_{\min}}{D_{f}-1}. \end{array}$$

Therefore, Xu and Yu [36] on the base of geometrical considerations give the following relation for the ratio $L_{0}/\bar{\lambda }$:

(12) $$\begin{array}{c} \frac{L_{0}}{\bar{\lambda }}=\frac{D_{f}-1}{D_{f}^{1/2}}{\left[\frac{1-\varphi }{\varphi }\frac{\pi }{4\left(2-D_{f}\right)}\right]}^{1/2}\frac{\lambda_{\max}}{\lambda_{\min}}. \end{array}$$

By this last relation it is possible to define the tortuosity dimension, *D*_*T*_, expressed by (9) and then fall back on the hydraulic conductivity law defined by the relation (6).

The evaluation of the lower and upper cut-off limits within the relationships (4), (9), and (12) and in the same relation (6) is not of immediate determination, because there is an implicit dependence of the porosity on the values of *λ* and in particular on the use of the relationship (8) as previously highlighted. This problem is more evident in the experimental measurements that show discrepancies already highlighted by Muller and McCauley [41] regarding the investigations conducted by Jacquin and Adler [12] and partially addressed by Xu and Yu [36] in the context of their theoretical and experimental investigations. In this framework the role of the measurement scale and the structure of the porous medium, and then the characteristic parameters influenced altogether by the medium heterogeneity locally or globally, are of fundamental importance for the choice of fractal models to use. In fact, this choice is also determined by potential simple and multiscaling behaviours of the same hydraulic conductivity, as well as the functional link *φ* = *φ*(*s*, *λ*) between the porosity, the measurement scale (*s*), and the pore size (*λ*).

Therefore, in this work it was deemed appropriate to proceed on the basis of simple-scaling considerations, evaluating the fractal behaviour of the hydraulic conductivity and effective porosity and comparing the models introduced by the above-cited authors and relation (3), which provides an immediate description of the experimental trend of the parameter considered for the aquifer under investigation and the values found by laboratory and field measurements, while retaining valid the self-similarity assumptions for the measurement scales observed.

## 3. Experimental Data Setting

In the present work the relationship (4) between hydraulic conductivity and effective porosity was experimentally verified [10, 13, 14, 17, 20, 57]. For this purpose values of *k* and *φ* obtained by both field and laboratory measurements were taken into consideration, because the values of these parameters and their spatial variation do not depend on the specific method of measurement [8], but on the aquifer volume involved [58].

**Figure 1:**
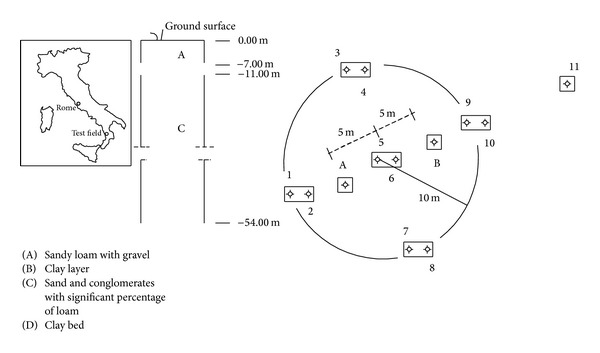
Stratigraphical and planimetrical layout of the test field.

The field measurements were carried out on the confined aquifer of Montalto Uffugo (Italy) test field. This area has the geological characteristics of a recently formed valley, with conglomeratic and sandy alluvial deposits. Corresponding to the test field, after a sandy surface layer with a thickness of about 7 m, one meets a clay lens with 4 m of thickness and then a layer of sand and silt depth up to 55 m, where a bank of consolidated clay starts. The test field has eleven wells and two piezometers. The wells marked with odd numbers affect the aquifer under pressure, below the clay layer, and of these only well number 11 is completely penetrating, while the others reach 40 m in depth. The two piezometers A and B are both entirely penetrating in the confined aquifer. A stratigraphic and planimetrical layout of the test field area is shown in Figure 1.

A total of 67 values of *k* and *φ* were measured, 5 of these by tracer tests, 15 by slug tests, and other 15 by aquifer tests.

The tracer tests were performed all in forced flow conditions, using number 1 as the tracer inflow well and number 5 as the pumping and observation well. These two wells are 10 m apart. For all the tests NaCl was used as the tracer in well number 1 in a solution volume of 0.4 m^3^, with an NaCl concentration of 200 kg/m^3^. The tracer inflow was performed in a short time for each test. The pumping rates were held constant during each tracer test considered, while the respective durations ranged between 5.4 and 34.84 days. The steady state conditions of the aquifer flow were verified for each tracer test and the drawdown-times data were analyzed by the Dupuis method (1863) to determine *k*. Moreover, the velocity of Darcy (*V*_*D*_) [LT^−1^], the correspondent effective velocity (*V*) [LT^−1^], and the effective porosity (*φ*) were determined [59].

All the slug tests were carried out following the guidelines suggested by Butler et al. [60] and Butler [61]. Therefore these were performed only on well number 11 and on piezometers A and B, all completely penetrating. The water volumes *V* rapidly admitted in the columns during the tests ranged between 0.003 m^3^ and 0.040 m^3^ and the water level variations were measured by proper pressure transducers at fixed times [61]. Once the geometry of the system aquifer well is known, to determine *k* and *φ*, the drawdown-time data sets obtained in this way were analyzed by the Cooper method [62].

For the aquifer tests, carried out in unsteady state conditions, the drawdown-time data were analyzed by the Neuman [63] and Jacob [64] methods, considering the initial and boundary conditions and the geometry of the system well known and taking into account that during the pumping the aquifer behaviour passed from confined to phreatic, because the aquifer proves to be under weak pressure. All the tests were performed to a constant pumping rate between 5.7 · 10^−4^ m^3^/s and 4.55 · 10^−3^ m^3^/s and for time ranges between 23 and 94.8 hours. In this way it was possible to determine the hydraulic conductivity (*k*), the storage coefficient (*S*), and the effective porosity (*φ*).

The laboratory measurements were carried out on 32 undisturbed soil samples, 18 of which were drawn out from the drilling column of piezometer A and number 14 from that of piezometer B, at several depths, between 11 m and 55 m from the ground surface. The hydraulic conductivity was measured for each of these samples, using flow cells as permeameter, and the effective porosity by the *double weighting* method. Further details about the measurement methodologies can be found in previous works [11].

## 4. Results and Discussion

The first step in the analysis of the hydraulic conductivity behavior as a function of porosity concerned the grouping of all the data sets obtained by the above-mentioned measurement methods.

The analysis of the experimental data highlights the scalar behavior of the hydraulic conductivity and effective porosity, albeit in a more evident manner for the first parameter and less marked for the second. However, for the analysis of the trend of these parameters with the scale, one can refer to previous studies relating to the same aquifer of the Montalto Uffugo test field [11].

Considering that both parameters *k* and *φ* are functions of the scale *s*, it is well known that there is a direct link between these two quantities; see, for example, the empirical relationships in the context of grain size distribution [45, 46], or in some fractal patterns [22]. Therefore, taking into account this analysis, at the laboratory scale pairs of values (*k*, *φ*) were obtained, determined by the spatial variability of the sampling point in the thickness of the aquifer, along the vertical drillings of the piezometers considered. However, given that these measures are used in a context in which field measurements are also considered and because they provide values of *k* and *φ* averaged on the entire volume of the aquifer involved in the measurement, it was deemed appropriate, also for the measurements performed in the laboratory, to consider the corresponding mean values, resulting, respectively, 3.25 · 10^−7^ m/s for *k* and 2.37 · 10^−2^ for *φ*.

Similarly to what was performed for *k* and *φ* values measured in the laboratory, even for those obtained by slug tests, the mean values were considered for each data set relative, respectively, to piezometers A and B and to well number 11 (see Figure 1), showing different geometrical characteristics. For *k* these mean that values were, respectively, found equal to 2.53 · 10^−6^ m/s for piezometers A and B and 2.66 · 10^−6^ m/s for well number 11, while for *φ* the corresponding values were found equal to 5.44 · 10^−2^ for piezometers A and B and 5.77 · 10^−2^ for well number 11.

The *k* values obtained by tracer tests ranged between 1.83 · 10^−6^ m/s and 6.00 · 10^−6^ m/s, while the correspondent *φ* values are in the range bounded by 4.50 · 10^−2^–8.26 · 10^−2^.

Similarly, the *k* values measured by aquifer tests ranged between 3:28 · 10^−6^ m/s and 5.78 · 10^−6^ m/s, while the correspondent *φ* values are in the range bounded by 6.25 · 10^−2^–9.77 · 10^−2^.

**Figure 2:**
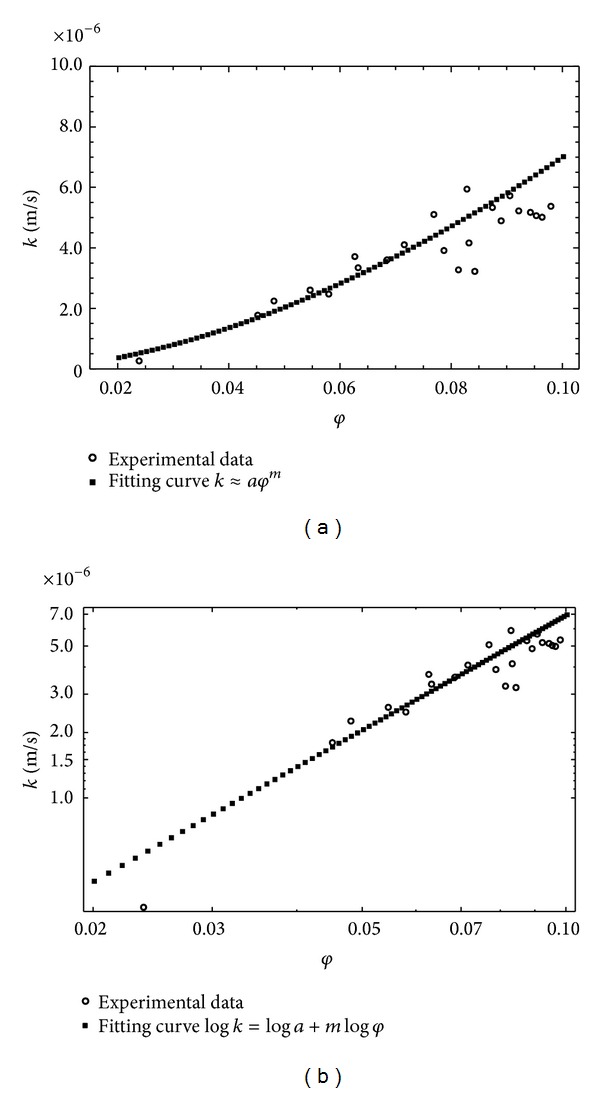
Trend of *k* versus *φ*_*e*_(*s*) according to (4): (a) decimal scale; (b) logarithmical scale.

Therefore, the scaling analysis relative to the values *k*[*φ*(*s*)] was carried out, considering the relation (3) and estimating the scale parameters corresponding to this power law. In this case, relatively to all measurement scales taken into consideration, the value of the scale index *m* was 1.753, while the coefficient *a* was 0.0004, with a coefficient of determination of the interpolation law *R* ^2^ = 0.887, with a value of the root mean square error (RMSE) 8.903 · 10^−7^. Figures 2(a) and 2(b) show the trends of the hydraulic conductivity evaluated as a function of the effective porosity, as well as the interpolation law of the power type corresponding to (4). Specifically Figure 2(b) show a scaling in which it is possible to observe the absence of cut-off limits and therefore to consider this behavior as a simple-scaling, allowing the self-similarity properties to be extended to the whole aquifer. For this reason the use of the models examined here is acceptable.

Furthermore, the trend of *k* was also described by relation (1), taking as a coefficient *μ* the relations proposed by Jacquin and Adler [12] and Muller and McCauley [41].

**Figure 3:**
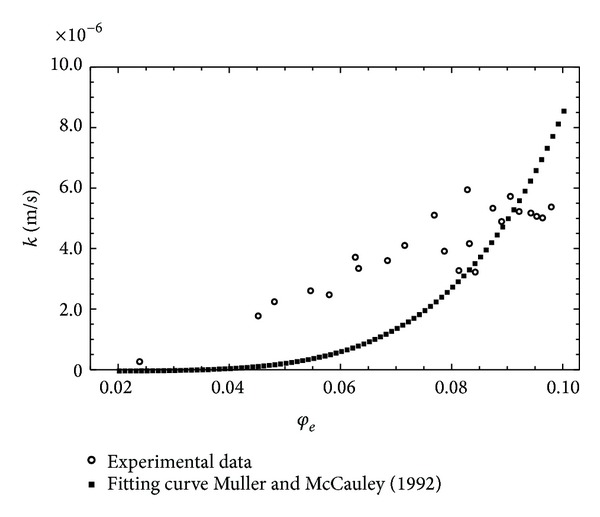
Fitting curve based on the Muller and McCauley [41] model.

**Figure 4:**
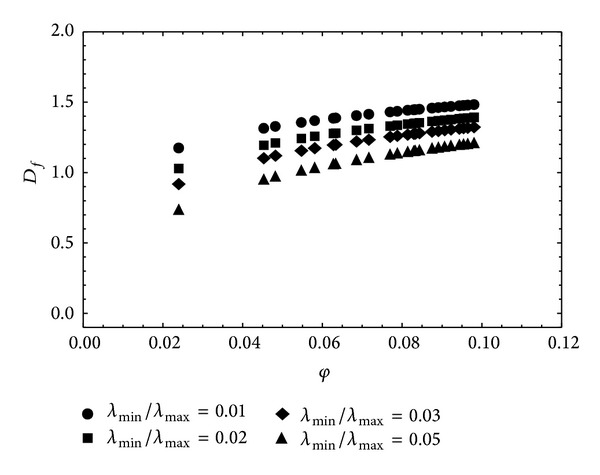
Relationship between fractal dimensions and porosity for different ratios *λ*_min⁡_/*λ*_max⁡_ according to Xu and Yu [36] model.

In this case the nonlinear fitting procedure allowed the fractal dimension values to be determined for each of the proposed relationships. According to the first model, the value of the *μ* index was 5.066, with a value of the fractal dimension 1.508 and standard error 0.0051. Considering the second model, the value of the *μ* index remained almost unchanged, while that of the fractal dimension was 0.659, with a standard error value 0.0045. For both models, the value of RMSE was 1.839 · 10^−6^, namely, greater than that obtained using relationship (3). As an example, Figure 3 shows the fitting curve relative to the Muller and McCauley [41] model; this curve is almost coincident with that relative to the Jacquin and Adler [12] model. On the basis of the results obtained it can be said that the direct use of (3) allows, without the constraints resulting from the presence of the fractal dimension in the exponent *μ*, a direct estimation of the general scale crowing index *m* with a smaller value of RMSE. In order to investigate the link between the general scales crowing index and the fractal dimension, the model proposed by Xu and Yu [36] was considered, taking into account relation (6), according to which the hydraulic conductivity proves to be a function, besides of the effective porosity, of the fractal dimensions and tortuosity, expressed, respectively, by relations (5) and (9). One should consider that in the relationship proposed by Yu and Li [47] the link between the effective porosity and the fractal dimension is based exclusively on the determination of the minimum and maximum values of the pore diameters characterizing the saturated porous medium. Relation (5) provides, in fact, on the basis of relation (4), a parametric relationship in which *λ*_min⁡_ and *λ*_max⁡_ are in constant ratio according to the porous medium under consideration. In the present case four different values of the ratio *λ*_min⁡_/*λ*_max⁡_, that is, 0.01, 0.02, 0.03, and 0.05, were considered (see Figure 4). The peculiarity of the Xu and Yu [36] method allows in any case, on the basis of the grain size distribution, determination of the geometrical characteristics relating to the maximum pore diameter, as expressed by relation (8), and then determination, on the basis of geometric considerations [36], of the ratio $L_{0}/\bar{\lambda }$, all in consideration of relations (11) and (12), which are functions only of the fractal dimension. On other hand, regarding the estimation of the tortuosity dimension according to (9), this proves to be dependent, in addition to the aforementioned ratio *λ*_min⁡_/*λ*_max⁡_, even on the average tortuosity as expressed by relation (10). Therefore this step is of crucial importance, on the basis of relationships (11) and (12), to assign the upper and lower limits of self-similarity in the scale of *φ*(*s*, *λ*), then to characterize precisely the *λ*_max⁡_ value expressed by (8) on which essentially the model of Xu and Yu [36] is based.

**Figure 5:**
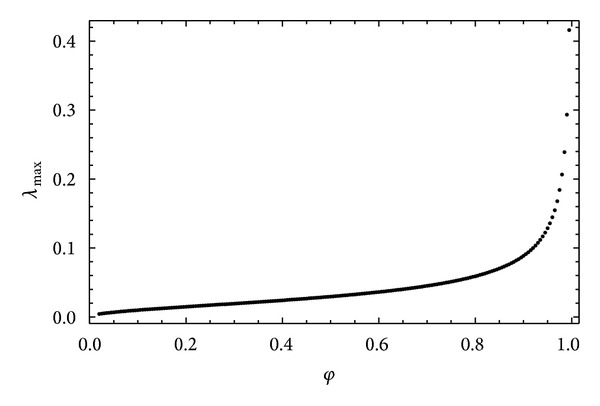
: Variation law of *λ*_max⁡_ versus *φ* for *d* = 0.041.

The experimental evidence in this case of sandy loam soil showed that for relationship (8) the value of *λ*_max⁡_ must be researched in a range of porosity values exceeding those measured with the purpose of the convergence of the Xu and Yu [36] model. As regards the parameter *d* a value was assumed of 0.041 mm, obtained as the maximum of the *d*_10_ values of the various soil samples extracted from the drilling columns of piezometers A and B and analyzed in the laboratory, assuming the particle size as effective grain diameter, of which 10% of the sample is finer. This assumption is justified because parameter *d* affects proportionally the variation law of *λ*_max⁡_, represented in Figure 5, which shows two different variation modes of this parameter with the porosity, that is, two different slopes of the representative curve. Furthermore, this curve represents the distribution law of *λ*_max⁡_ as a function of the porosity. Therefore, recalling (8), in consideration of the following integral:

(13) $$\begin{array}{c} \overset{1}{\underset{0}{\int }}\lambda d\varphi =\overset{1}{\underset{0}{\int }}d\sqrt{\frac{\varphi }{1-\varphi }}d\varphi =\frac{d\pi }{2}, \end{array}$$

it is possible to estimate the value of *d* to be assumed in the Xu and Yu [36] model taking into account the proportionality constant *π*/2.

**Figure 6:**
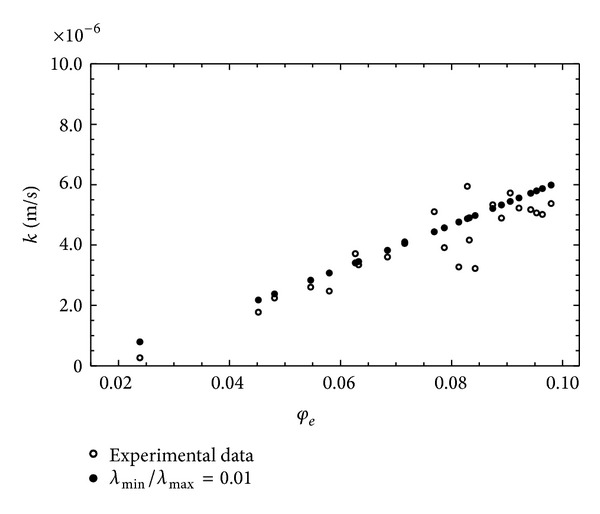
Fitting law according to the Xu and Yu [36] model for *λ*_min⁡_/*λ*_max⁡_ = 0.01.

**Figure 7:**
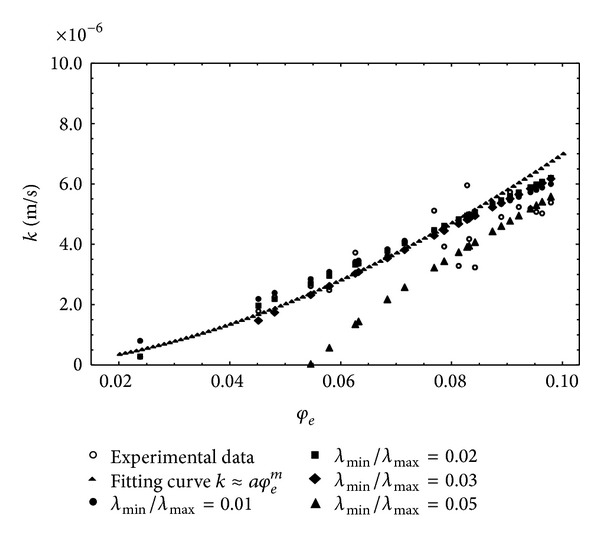
Fitting curves: for experimental and Xu and Yu [36] models.

Indeed, in this case the *d* value, determined by relation (13), is equal to 0.041, which is coincident with the maximum value of *d*_10_, also given above. The value of the integral to the left in (13) represents the area under curve *λ*_max⁡_ − *φ* and therefore allows approximate estimation of the value of *λ*_max⁡_, corresponding to about 50% of this area, which in this case is 0.13 and this can be easily verified by the integral mean value theorem. This value allowed more accurate utilization of the Xu and Yu [36] model for a value of the rate *λ*_min⁡_/*λ*_max⁡_ of 0.01. Regarding this ratio the fractal dimension value is next to 1.4–1.5, as well as close to that estimated by the Jacquin and Adler [12] model.  Figure 6 shows this interpolation law assessed on the basis of the experimental data. The RMSE value corresponding to this law is 6.984 · 10^−7^, according to the data of Table 1, which is the lowest and closer to the experimental one obtained by (3).  Figure 7 shows all the representative curves obtained by the Xu and Yu [36] model to vary the ratio *λ*_min⁡_/*λ*_max⁡_ in the range 0.01–0.05, with the experimental values and the experimental fitting law represented by (3).

It should be pointed out that, on the basis of the experimental results, relation (3) gives in any case the opportunity to characterize the hydraulic conductivity behavior in a simple way and with sufficient reliability. This result allows the characteristic scale parameter to be obtained in a direct manner, without the use of a fractal model, which in any case requires the knowledge of a larger number of parameters. It is also noted that relation (3) gives an RMSE value less than that given by the models of Jacquin and Adler [12] and Muller and McCauley [41] and slightly higher than that of the Xu and Yu [36] model (see Table 1).

The methodology shown here, based essentially on the coupled use of relationship (3) and the Xu and Yu [36] model, may find a general use, extendable also to other soil types, while the values of the parameters *μ* and *m* here obtained can be reasonably taken into consideration for soils belonging to the same class as that examined here.

## 5. Conclusions

The analysis of the direct scaling of the hydraulic conductivity and effective porosity was performed for a confined aquifer made up of a sandy-loam soil, considered homogeneous owing to the absence of stratifications. The measurements regarded the scale size of the parameters in relation to the laboratory, small, medium, and large field.

Based on the measurements carried out, the representativeness of the law of scaling was sought, according to both the scale and the functional link between the hydraulic conductivity and effective porosity, highlighting the simple-scaling behavior, without considering however the high resolution field. This simplification, however, does not show in any case a trend representable by a multiscaling behavior.

The analysis of the scale index, obtained by appropriate laws of power type characterizing the aquifer taken into consideration, allows information about the fractal dimensions to be given indirectly in order to estimate using other specific models, considering only the physical scaling quantities, which, as is well known, are closely connected to the aforementioned power laws.

In this work a comparison between the experimental scaling law and some fractal models present in the literature was also considered. Specifically, the models of Jacquin and Adler [12], Muller and McCauley [41], and Xu and Yu [36] were considered. The results obtained showed a high degree of reliability of the experimental model represented by relation (3), compared to other models examined.

In fact, this power law model by direct scaling produced a value of the root mean square error smaller than that of the fractal models of Jacquin and Adler [12] and Muller and McCauley [41] and of the same order of magnitude as the fractal model of Xu and Yu [36]. About the use of this last model the behaviour of the variability of the largest diameter *λ*_max⁡_ value was analysed as a function of the porosity measured. Specifically, greater details were provided about the value of this parameter to use for a given soil.

The model represented by relation (3) allows definition of the relationship between the variables under consideration in a simpler manner than that of the fractal models mentioned above, because it requires the consideration of a smaller number of parameters. In any case, using this approach in a coupled way with the model of Xu and Yu [36] can be advantageous, even in soils of a different type from the one considered here.

## Figures and Tables

**Table 1:** Main parameter values of the fractal models analyzed.

Models	*μ*	*D*_*f*_	RMSE
Experimental power law	1.753	—	8.903 · 10^−7^
Jacquin and Adler (1987) [12]	5.066	1.508	1.839 · 10^−6^
Muller and McCauley (1992) [41]	5.066	0.659	1.839 · 10^−6^
Xu and Yu (2008)* [36]	—	1.4-1.5	6.984 · 10^−7^

*RMSE value is evaluated for *λ*_min⁡_/*λ*_max⁡_ = 0.01.
